# Lower mortality rates in those living at moderate altitude

**DOI:** 10.18632/aging.101057

**Published:** 2016-10-02

**Authors:** Martin Burtscher

**Affiliations:** Department of Sport Science, Medical Section, University of Innsbruck, Austria

**Keywords:** altitude, mortality, cancer, cardiovascular disease

Individuals living at moderate altitudes (up to about 2000 m) were shown to have lower mortality from coronary artery disease (CAD) and stroke (−22% and −12% per 1000 m) [1] and an about 50% lower risk of dying from Alzheimer's disease compared with their counterparts living at lower altitudes [2]. In contrast, reported altitude effects on cancer mortality are still conflicting [3]. However, due to shared risk factors, e.g. obesity and diabetes, in cardiovascular disease and cancer a shared biology for both disease entities may be assumed [4]. Therefore, it is hypothesized that mortality from certain cancers will decline with increasing altitude as demonstrated for CAD.

Altitude-dependent mortality from CAD, male colorectal cancer and female breast cancer from 2003 to 2012 in Austria has been evaluated based on data from the Austrian Mortality Registries (Statistik Austria) [5]. Since the phenomenon of migration was most pronounced towards larger communities (>20,000 population) [5] only communities with a population below 20,000 were included to avoid important confounding from migration. Effects of more rural conditions have at least partly been evaluated by considering agriculture employment (<3%, 3-7%, and >7%). The total numbers of deaths amounted to 87,127 from CAD (ICD-10: 120-125), 7,640 from male colorectal cancer (ICD-10: C18-C21), and 8,953 from female breast cancer (ICD-10: C50). Age-standardized mortality rates (ASMR) per 100,000 population and 95% confidence intervals (CI), based on the assumption that the data follow a Poisson distribution, are reported (provided by Statistik Austria) [5].

**Figure 1:**
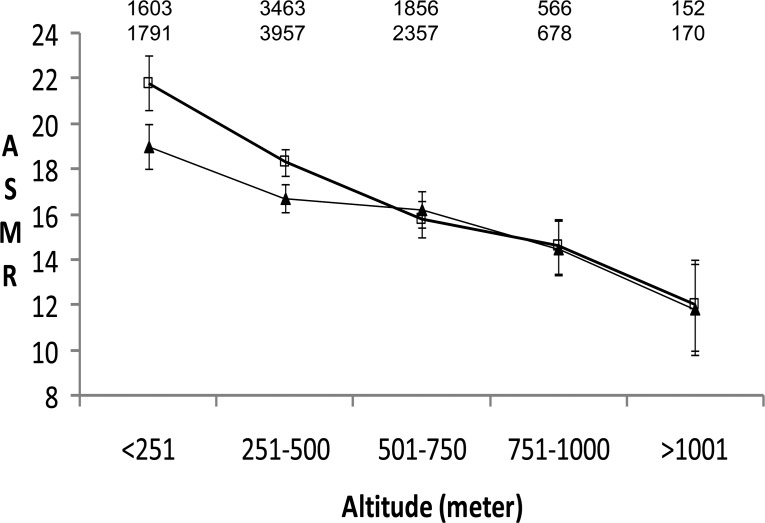
Altitude-dependent decrease of age-standardized mortality rates (ASMR, 95% CI) from male colorectal cancer (squares) and female breast cancer (triangles). The numbers indicate N of deaths from male colorectal cancer (upper line) and from female breast cancer (lower line) at different altitudes.

The general life expectancy, e.g. in 2009, increased from low altitude (<251 m) to higher altitudes (1001 to about 2000 m) by about 2 years, in males from 76.7 to 79.1 years and in females from 82.1 to 84.1 years [5]. From low (<251 m) to higher (1001 to about 2000 m) altitudes, ASMR (95% CI) from CAD decreased by 28% (21.7 − 34.1%) from 106.5 (104.2 − 108.9) to 76.7 (71.8 − 81.6) in males and by 31% (24.5 − 37.4%) from 56.8 (55.4 − 58.2) to 39.1 (36.4 − 41.8) in females. ASMR from male colorectal cancer and female breast cancer decreased almost linearly from low to higher altitude by 45% and 38% (Figure 1). Independent of altitude, increasing agriculture employment was associated with a diminished ASMR from ischemic heart disease by about 15% for males and females [5]. In contrast, solely increasing altitude was related to the reduction in cancer mortality.

The lower mortality from CAD at moderate altitudes is in close agreement with that reported from Switzerland [1]. Reduced oxygenation at higher altitudes and altitude-related climate changes, e.g. temperature, UV-radiation, and/or air-pollution but also differences in dietary behaviour were considered as potentially protective factors [1]. Similarly, a set of altitude-dependent environmental and life-style factors have been suggested to contribute to lower mortality from Alzheimer's disease at higher altitudes [2]. The present data extend preventive effects of living at higher elevations on male colorectal and female breast cancer mortality. The observation that more rural conditions may not have affected cancer mortality will even heighten the importance of altitude-specific effects. The nearly linear mortality reduction with increasing altitude strongly indicates a dose-response relationship. Unfortunately, to date only little and conflicting information is available on cancer mortality at altitude [3, 6]. Given the fact of shared risk factors in cardiovascular disease and cancer [4] the beneficial effects of moderate hypoxia stimuli at altitudes up to 2500 m on cardiovascular risk factors [3] might also contribute to the lowering of cancer mortality. For instance, obesity and diabetes are such shared risk factors which have recently been reported to be lower in US individuals living at higher altitudes [7]. These authors speculated that cold-induced thermogenesis, decreased appetite, unintentional increased physical activity, and hypoxia-related better glucose tolerance could represent potential mechanisms explaining the inverse relationship between the prevalence of obesity and/or diabetes and altitude [7]. The author of an ecological study attributed lower cancer death rates at higher places to elevated natural background radiation (hormesis theory) but emphasized that causal inferences cannot be made [6]. Besides changing climate conditions with increasing altitude a potentially higher exercise capacity in the altitude population helps to explain lower mortality [8]. Whereas high-altitude regions like Leadville in Colorado (US) or the Altiplano in South America are rather flat, in the Alps the amount of hilly terrain increases steeply with altitude likely contributing to a higher fitness level in the altitude population. In the Swiss study a similar amount of physical activity in the low and higher altitude populations has been suggested [1]. However, since the hilly terrain is much more challenging than the plain terrain, e.g. when walking or cycling, a similar amount of physical outdoor activities can result in higher exercise capacity in the altitude population [9]. In any case, the remarkable protective effects of living at moderate altitudes also on cancer mortality are fascinating and deserve further investigation.
